# Heterogeneous Response of Tumor Cell Lines to Inhibition of Aspartate β-hydroxylase

**DOI:** 10.7150/jca.94452

**Published:** 2024-04-29

**Authors:** Madiha Kanwal, Ingrid Polakova, Mark Olsen, Murtaza Khan Kasi, Ruth Tachezy, Michal Smahel

**Affiliations:** 1 Department of Genetics and Microbiology, Faculty of Science, Charles University, BIOCEV, Vestec, Czech Republic.; 2 Department of Pharmaceutical Sciences, College of Pharmacy - Glendale, Midwestern University, Glendale, AZ, USA.

**Keywords:** ASPH inhibitors, tumorigenesis, Notch pathway, AKT signaling, heterogeneity, cell cycle

## Abstract

**Background**: Cancer development involves alterations in key cellular pathways, with aspartate β-hydroxylase (ASPH) emerging as an important player in tumorigenesis. ASPH is upregulated in various cancer types, where it promotes cancer progression mainly by regulating the Notch1 and SRC pathways.

**Methods**: This study explored the responses of various human cervical, pharyngeal, and breast tumor cell lines to second- and third-generation ASPH inhibitors (MO-I-1151 and MO-I-1182) using proliferation, migration, and invasion assays; western blotting; and cell cycle analysis.

**Results**: ASPH inhibition significantly reduced cell proliferation, migration, and invasion and disrupted both the canonical and noncanonical Notch1 pathways. The noncanonical pathway was particularly mediated by AKT signaling. Cell cycle analysis revealed a marked reduction in cyclin D1 expression, further confirming the inhibitory effect of ASPH inhibitors on cell proliferation. Additional analysis revealed G0/G1 arrest and restricted progression into S phase, highlighting the regulatory impact of ASPH inhibitors on the cell cycle. Furthermore, ASPH inhibition induced distinctive alterations in nuclear morphology. The high heterogeneity in the responses of individual tumor cell lines to ASPH inhibitors, both quantitatively and qualitatively, underscores the complex network of mechanisms that are regulated by ASPH and influence the efficacy of ASPH inhibition. The effects of ASPH inhibitors on Notch1 pathway activity, cyclin D1 expression, and nuclear morphology contribute to the understanding of the multifaceted effects of these inhibitors on cancer cell behavior.

**Conclusion**: This study not only suggests that ASPH inhibitors are effective against tumor cell progression, in part through the induction of cell cycle arrest, but also highlights the diverse and heterogeneous effects of these inhibitors on the behavior of tumor cells of different origins.

## Introduction

Aspartate β-hydroxylase (ASPH) has been identified as an essential player in the regulation of several cellular pathways associated with tumorigenesis 1. ASPH functions as a type II transmembrane hydroxylase and belongs to the highly conserved α-ketoglutarate-dependent hydroxylases. This enzyme plays a pivotal role in the post-translational hydroxylation of aspartyl and asparaginyl residues in epidermal growth factor (EGF)-like structural domains of various proteins such as Notch receptors and their ligands 2-4. ASPH is upregulated in various malignant neoplasms; it has been detected in 70-90% of human solid tumors, where it plays a crucial role in mediating a malignant phenotype characterized by increased cell proliferation, invasion, and metastasis 5-8.

Dysregulation of the Notch1 pathway can be implicated in carcinogenesis 8,9. ASPH-mediated upregulation of Notch1 signaling by hydroxylation of Notch1 and its ligands has been detected in various cancer types, such as hepatocellular carcinoma (HCC) 8, pancreatic cancer 10, and breast cancer 11. The canonical Notch1 pathway is activated when Delta, Serrate, or Lag-2 (DSL family ligands) bind to the Notch1 receptor on neighboring cells. This interaction leads to the initiation of a series of proteolytic cleavages mediated by a disintegrin and metalloproteinase (ADAM) and γ-secretase enzymes, resulting in the production of the cleaved Notch intracellular domain (NICD) fragment, its transport to the nucleus, and upregulation of target genes, including the hes family Bhlh transcription factor 1 (HES1), the c-myc proto-oncogene, and cyclin D1 9,12. In addition to the canonical pathway, Notch signaling can also be mediated by a noncanonical pathway involving NICD interaction with nuclear factor κB (NFκB), phosphatase and tensin homolog (PTEN), protein kinase B (AKT), Hippo, transforming growth factor β (TGF-β), Wnt, and mechanistic target of rapamycin kinase C2 (mTORC2) pathways in cytoplasm and/or nucleus, and regulating biological processes such as cell survival, metabolism, and differentiation 12. The interaction between ASPH, Notch1, and AKT provides an intriguing opportunity for investigating new therapeutic approaches.

In this regard, targeting ASPH has received considerable attention. Several small molecule inhibitors (SMIs) have been designed as potential inhibitors of ASPH β-hydroxylase activity based on the crystal structure of the catalytic site in the C-terminus of ASPH 8,13. Derivatives of the MO-I-1100 inhibitor, second-generation MO-I-1151 (with a trifluoromethyl group substitution instead of chlorine) 14, and third-generation MO-I-1182 (in which the trifluoromethyl group was replaced with a carboxymethyl group) 15, efficiently enhanced the potency of ASPH inhibition in *in vitro* assays of tumor cell proliferation, migration, and invasion. For MO-I-1100, 80% reduction of enzymatic activity has been reported 8. However, it is unclear whether the substitutions in MO-I-1151 and MO-I-1182 inhibitors increased inhibitory activity or just enhanced solubility parameters. Several animal tumor models have also demonstrated anti-tumor effects of selective inhibition of ASPH activity 8,10,14-17. These findings highlight the potential of SMIs as effective tools for inhibiting ASPH-mediated cellular processes, which represents a promising avenue for cancer therapeutic development.

In this study, we investigated the intricate interaction between ASPH and the Notch1 signaling pathway in various cancer cell lines. Notch1 signaling is a major pathway involved in cancer progression that is influenced by ASPH, but the underlying mechanisms of the influence of ASPH on the noncanonical pathway involved in this interaction have not been determined. Recognizing this gap in the current knowledge, our investigation not only addressed it by assessing the effects of second-generation (MO-I-1151) and third-generation (MO-I-1182) ASPH inhibitors on both the canonical and noncanonical Notch1 pathways but also stands as the first to explore the effects of these inhibitors in the context of cervical and pharyngeal cancers. By shedding light on previously unexplored cancer types, our study revealed the complex nature of ASPH-mediated Notch1 pathway regulation, but more importantly, highlighted the heterogeneous response of different cancer types to ASPH inhibition.

## Materials and methods

### Cell culture

The cell lines used in this study were derived from a variety of carcinoma types. HeLa cells (HPV18-positive cervical epithelial adenocarcinoma), FaDu cells (HPV-negative hypopharyngeal carcinoma), Detroit 562 cells (HPV-negative pharyngeal carcinoma), CaSki cells (HPV16-positive cervical squamous cell carcinoma), and SiHa cells (HPV16-positive cervical squamous cell carcinoma) were obtained from the American Type Culture Collection (ATCC; Manassas, VA, USA). MCF-7 cells (HPV-negative breast carcinoma) were kindly provided by Dr. Libor Macurek (Institute of Molecular Genetics, Czech Republic).

HeLa, CaSki, and SiHa cells were cultured in Roswell Park Memorial Institute 1640 (RPMI-1640) medium (Sigma‒Aldrich, St. Louis, MO, USA); FaDu and Detroit 562 cells in Eagle's minimum essential *medium* (EMEM; Sigma‒Aldrich); and MCF-7 cells in Dulbecco's modified Eagle's medium (DMEM; Sigma‒Aldrich). All cell culture media were supplemented with 10% fetal bovine serum (FBS; Sigma‒Aldrich), 100 IU/mL penicillin, and 100 μg/mL streptomycin (Biosera, Kansas, MO, USA). The cells were cultured in an incubator at 37°C in 5% CO_2_ and periodically checked for mycoplasma contamination. The cells were harvested at 80% confluency using a 0.05% trypsin-EDTA solution in phosphate-buffered saline (PBS) for further examination.

### MTT assay

To investigate the dose-dependent effect of ASPH inhibitors on cell proliferation, a 3-(4,5-dimethylthiazol-2-yl)-2,5-diphenyl tetrazolium bromide (MTT) assay was performed. Cells were seeded in 96-well plates at a density of 1×10^4^ cells per well. The concentrations of the ASPH inhibitors MO-I-1151 and MO-I-1182 dissolved in dimethyl sulfoxide (DMSO) ranged from 0.1 to 20 μM in all the cell lines. After 48 h of incubation, the culture media were replaced with growth media supplemented with MTT reagent (5 mg/ml) and incubated for 4 h. The resulting purple formazan crystals were subsequently dissolved in DMSO for 3 h, and the absorbance was measured at a wavelength of 562 nm using an Infinite M200 Pro microplate reader (TECAN, Mannedorf, Switzerland). The wells without cells but containing MTT reagent and DMSO were used to establish a baseline blank value, which was then subtracted from all the readings. The results are expressed as a percentage of the absorbance relative to the corresponding untreated control.

### Colony formation assay

To assess the potential of cancer cells to form colonies upon exposure to ASPH inhibitors, we used a clonogenic assay. Cells were seeded (in a 6-well plate 500 cells/well) and treated with the MO-I-1151 or MO-I-1182 inhibitors at a concentration of 20 μM. DMSO was used as a negative control. The treated cells were grown at 37°C for 7 days to facilitate colony formation. After incubation, the cells were thoroughly washed with PBS and fixed with 4% formaldehyde for 15 min. The cells were then stained with 0.05% crystal violet dye for 5 min. After gentle rinsing with water, images of each well were captured with a Brother MFC-J5320DW scanner (Brother, Nagoya, Japan) and analyzed using ImageJ software (www.imagej.org).

### 3D spheroid assay

To investigate the invasive potential of the cancer cells, spheroids were generated using sterilized 2D silicone molds (Sigma‒Aldrich) consisting of microwells filled with 2% agarose gel. After solidification, the agarose molds were transferred to a 12-well plate and then equilibrated with the respective culture media. Precultured cells were seeded into agarose molds and incubated for 48 h to form spheroids. To create a three-dimensional (3D) environment, a buffered solution consisting of the respective culture media, 0.375% (w/v) NaHCO_3_, 8.5 mM NaOH, and 15 mM HEPES was mixed at a 3:1 ratio with 1 mg/mL collagen solution (Cultrex 3-D Culture Matrix Rat Collagen I; R&D Systems, Minneapolis, MN, USA). This mixture was used as a matrix for embedding the spheroids. Culture media containing DMSO or 20 μM of MO-I-1151 or MO-I-1182 were layered after matrix polymerization. The embedded spheroids were photographed at 0 and 72 h, and the spheroid areas were determined using ImageJ software. At least three spheroids per condition were examined in each experiment.

### Wound healing assay

Cell migration was evaluated via a wound healing assay. Cells were seeded in 6-well plates. After 24 h of treatment with DMSO, MO-I-1151, or MO-I-1182 (20 μM), a scratch was made in the center of the wells using a sterile 200-μl pipette tip, followed by washing with PBS to remove cell debris. Cells were then incubated in specific media containing DMSO, MO-I-1151, or MO-I-1182. A Nikon-Eclipse TE2000-S microscope (Nikon, Tokyo, Japan) with a Hoffman modulation contrast (4× objective) was used to document cell migration toward the gap area at the designated location. ImageJ software was used to analyze the images taken at 0 and 12 h. The gap size at 12 h was calculated in comparison to the gap size at 0 h, and triplicates were averaged.

### Transwell migration and invasion assays

*In vitro* migration and invasion assays were conducted using uncoated and Matrigel-precoated transwell chambers (8 μm pore size; Corning, Lowell, MA, USA), respectively, in a 24-well plate. Briefly, 5×10^4^ cells from each cell line were suspended in their respective serum-free media and then seeded into the upper chamber. The cells were treated with MO-I-1151 or MO-I-1182 at a concentration of 20 μM. DMSO was used as a negative control. Medium supplemented with 10% FBS was used as a chemoattractant in the lower chamber. After incubating at 37 °C for 24 h, the noninvading cells were removed from the upper chamber with a cotton swab. The invading cells that passed through and adhered to the lower surface of the membrane were fixed with 95% ethanol for 15 min. Staining was performed with 0.05 % crystal violet staining solution for 5 min. Images were captured from 4 random fields of the membrane by an Olympus IX73 microscope (Olympus, Tokyo, Japan) using Hoffman modulation contrast (10× objective). The images from the triplicate experiments were examined using ImageJ software, and the cell density was estimated as the pixel intensity after background subtraction and contrast enhancement. The relative pixel intensity was calculated by dividing the mean pixel intensity of the image of the cells treated with MO-I-1151 or MO-I-1182 by that of the DMSO-treated sample.

### Immunoblotting

The cell lines were treated with either DMSO or 20 μM MO-I-1151 or MO-I-1182 for 24 h, and cells were lysed using a lysis buffer consisting of 62.5 mM Tris (pH 6.8), 20% glycerol, and 4% SDS (w/v). The protein concentration of each lysate was quantified using a BCA protein assay (ThermoFisher Scientific, Waltham, MA, USA). Equal amounts of lysate proteins were resolved in 10% sodium dodecyl sulfate-polyacrylamide gel electrophoresis (SDS‒PAGE) and then transferred to polyvinylidene fluoride (PVDF) membranes for immunoblotting. The membranes were blocked in 5% skim milk, followed by an overnight incubation with primary antibodies against ASPH (NBP1-69229, 1:1000; Novus Biologicals, Littleton, CO, USA and PB9478, 1:1000; Boster Biological Technology, Pleasanton, CA, USA); Notch1 (3608, 1:1000), activated Notch1 (NICD; 4147, 1:1000), HES1 (11988, 1:1000), Snail (3879, 1:500), Slug (9585, 1:500), Vimentin (5741, 1:1000), c-myc (18583, 1:1000), phospho-c-myc-Thr58 (46650), SRC (2109, 1:1000), pSRC-Tyr416 (6943, 1:1000), pGSK3β (9322, 1:1000) from Cell Signaling Technology (Danvers, MA, USA); GSK3β (SAB5300175, 1:500) from Sigma‒Aldrich; cyclin D1 (sc-8396, 1:1000, Santa Cruz Biotechnology; Dallas, TX, USA); AKT (680302, 1:500; BioLegend; San Diego, CA, USA); pAKT- pS473 (AB1645553; 1:1000) BD Biosciences, Franklin Lakes, NJ, USA); and GAPDH (MA5-157380, 1:2000; ThermoFisher Scientific). After washing with PBS containing 0.05% Tween 20, the membranes were incubated with horseradish peroxidase (HRP)-conjugated secondary antibodies for 2 h at room temperature. The protein bands were visualized using Immobilon ECL Ultra Western HRP Substrate (Millipore, Billerica, MA, USA) and detected with an Amersham Imager 600 (GE Healthcare, Chicago, IL, USA). To ensure equal loading of proteins, GAPDH was used as a loading control.

### Immunofluorescence microscopy

Cells were grown on coverslips. After 24 h of treatment with DMSO, MO-I-1151, or MO-I-1182 (20 μM), the cells were washed with PBS, pre-extracted with pre-extraction buffer (25 mM HEPES (pH 7.4), 50 mM NaCl, 1 mM EDTA, 3 mM MgCl_2_, 300 mM sucrose, 0.5% Triton X-100) for 5 min, fixed with 4% paraformaldehyde (PFA) for 15 min, and permeabilized with 0.2% Triton-X100 for 5 min. The cells were blocked with 1% bovine serum albumin (BSA) for 30 min and incubated with primary activated Notch1 antibody (ab8925, 1:50; Abcam, Cambridge, UK) for 2 h at room temperature, followed by incubation with Alexa Fluor 555 secondary antibody (A21429, 1:400; ThermoFisher Scientific) for 1 h at room temperature. The nuclei were stained with DAPI (FP1490, 1:500; Akoya Biosciences, Marlborough, MA, USA) for 5 min, and the coverslips were washed with distilled water and mounted onto glass slides with Fluoromount-G (4958-02; Life Technologies, Carlsbad, CA, USA). Immunofluorescence was analyzed with a 63× objective using an LSM 880 NLO confocal microscope (Carl Zeiss, Oberkochen, Germany), and images were processed using ImageJ software. Nuclear morphological changes were identified through examination of microscopic images, comparing the distinct nuclear features, including nuclear size and shape, between untreated and inhibitor-treated cells. The observed alterations in nuclear morphology were consistent across multiple microscopic fields.

### Cell cycle analysis

The cells were treated with MO-I-1151 or MO-I-1182 at a concentration of 20 μM for 48 h. The cells were harvested, washed with PBS, and fixed with ice-cold absolute ethanol for 15 min at -20°C. After centrifugation, the pelleted cells were rehydrated with PBS and stained with a staining solution consisting of 10 μg/ml propidium iodide (PI; P3566; ThermoFisher Scientific) and 200 μg/ml RNase A (EN0531; ThermoFisher Scientific) in Tris staining solution (100 mM Tris (pH 7.4), 100 mM NaCl, 1 mM CaCl_2_, 0.5 mM MgCl_2_, 0.1% NP-40) for 15 min at room temperature and subjected to cell cycle analysis using Cytoflex LX flow cytometer (Beckman Coulter, Indianapolis, IN, USA) and FlowJo software v10.9.0 (BD Biosciences).

### Statistical analysis

All the results are presented as the mean ± standard error of the mean (SEM). Prism 5 software (GraphPad, La Jolla, CA, USA) was used for graph generation and statistical analysis. Group comparisons were evaluated by t*-*test, and statistical significance was defined as a p-value <0.05.

## Results

### ASPH inhibitors reduce proliferation, migration, and invasion in human tumor cell lines with variable efficacy

**Figure 1:**
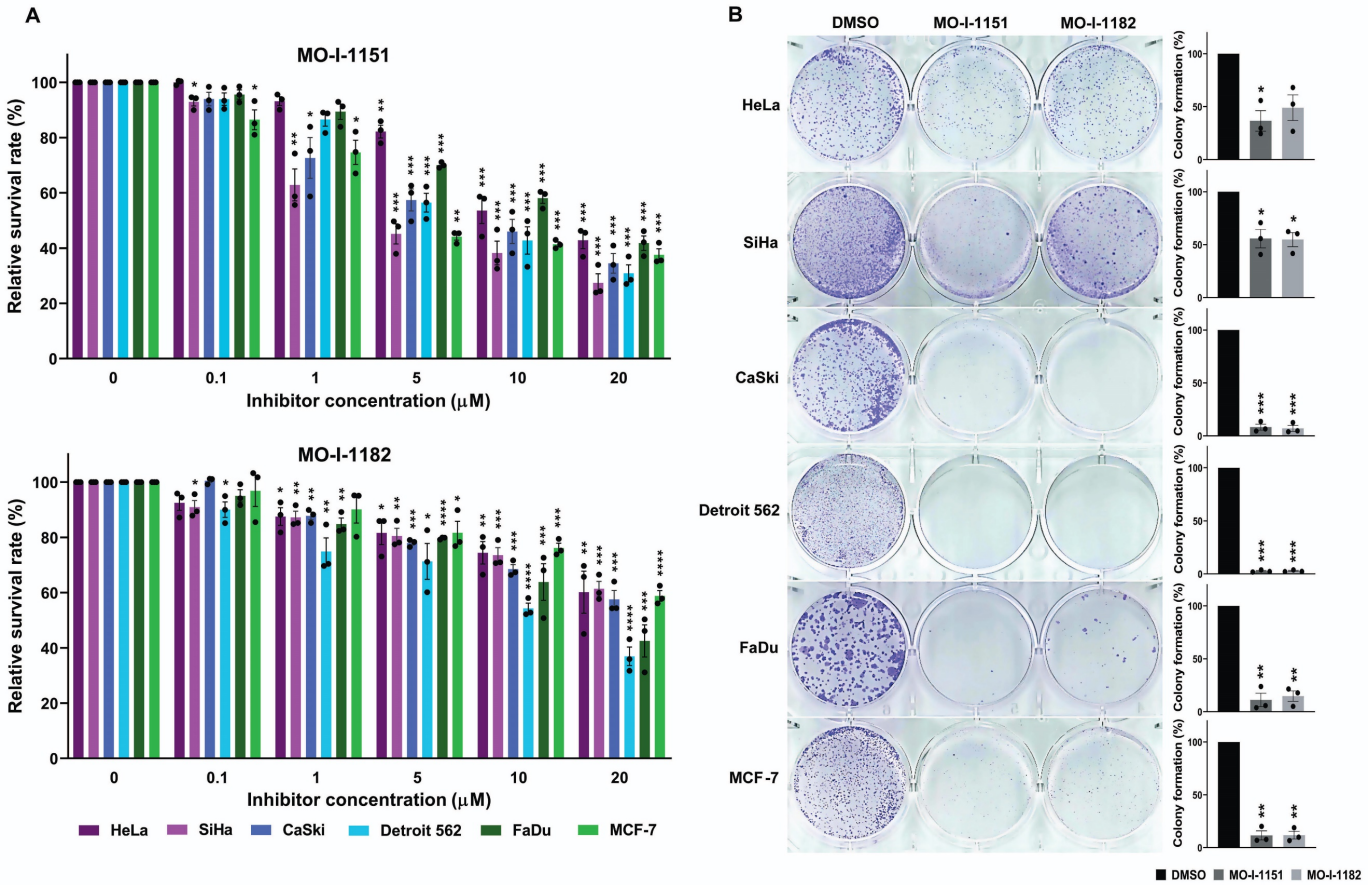
Effect of ASPH inhibitors on cell proliferation. **(A)** Cells were treated with MO-I-1151 and MO-I-1182 at concentrations of 0.1, 1, 5, 10, or 20 μM for 48 h, followed by an MTT assay. **(B)** Cells were incubated with 20 μM MO-I-1151 or MO-I-1182 for 7 days, stained with crystal violet, and imaged. Image quantification was performed using ImageJ software. DMSO was used as a control. The results are presented as the mean ± SEM of three independent experiments. The statistical significance refers to the comparison with the control (* p<0.05, ** p<0.01, *** p<0.001, **** p<0.0001).

To investigate the potential inhibitory effects of the ASPH inhibitors MO-I-1151 and MO-I-1182 on cell growth, we examined their effects on cell proliferation and clonogenic survival in a variety of human tumor cells. The experiments included cell lines derived from cervical (HeLa, SiHa, and CaSki), pharyngeal (FaDu and Detroit 562), and breast (MCF-7) cancers. These cell lines were treated with different concentrations of the inhibitors, and the proliferation inhibition rate was determined by an MTT assay. Both ASPH inhibitors were found to be potent suppressors of proliferation in all the tumor cell lines tested (Figure 1A). The inhibitory effect was dose dependent. Overall, MO-I-1151 showed more pronounced efficacy than MO-I-1182 against HeLa (IC_50_: 15.5 vs 26.2 µM), SiHa (IC_50_: 8.5 vs 27.3 µM), CaSki (IC_50_: 11.5 vs 22.1 µM), Detroit 562 (IC_50_: 11.1 vs 13.6 µM), FaDu (IC_50_: 15.2 vs 16.5 µM), and MCF-7 cells (IC_50_: 10.3 vs 24.6 µM).

To further investigate the effect of the MO-I-1151 and MO-I-1182 inhibitors on the proliferation of human cancer cells, we performed a clonogenic assay, which is based on the ability of a single cell to proliferate and form colonies. ASPH inhibitors at a concentration of 20 µM strongly reduced the colony-forming potential of CaSki, Detroit 562, FaDu, and MCF-7 cells, but these effects were lower in SiHa and HeLa cells (Fig. 1B). The inhibitors MO-I-1151 and MO-I-1182 had comparable effects on all the tumor cell lines tested.

**Figure 2:**
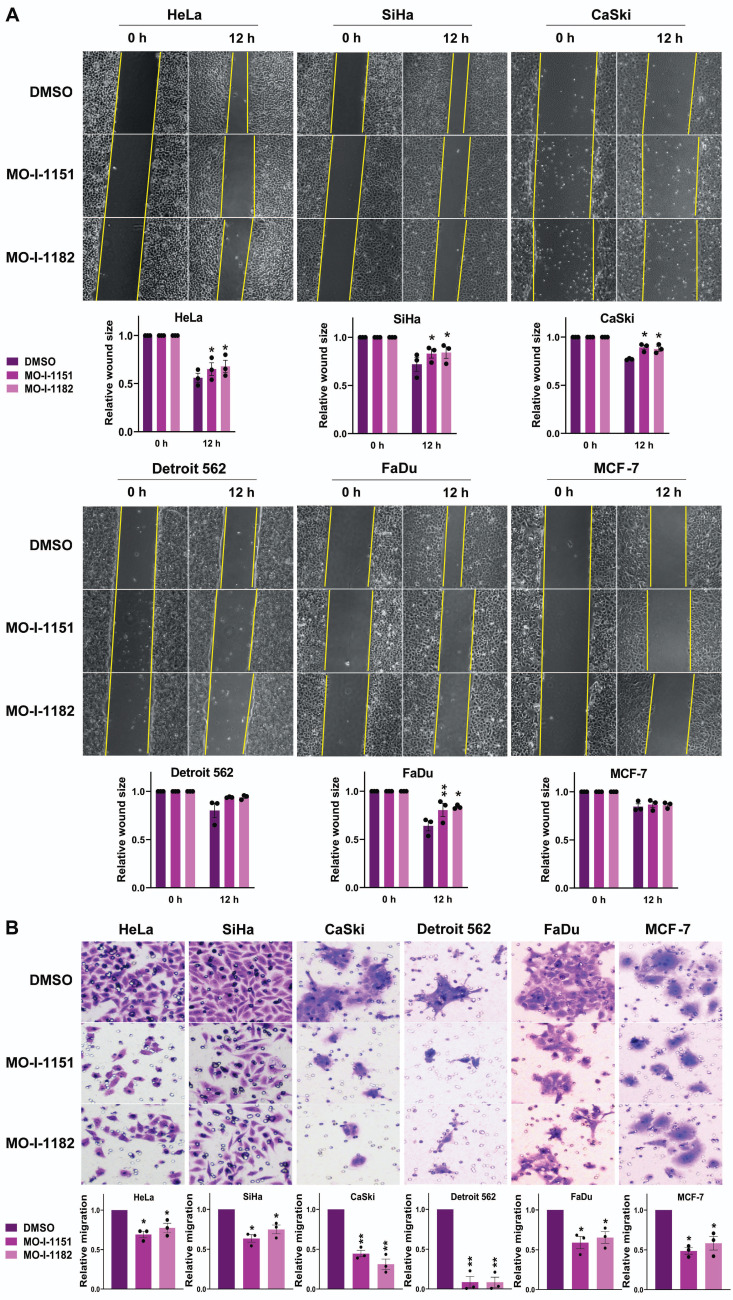
Effect of ASPH inhibitors on cell migration. **(A)** Cells were treated with 20 μM of the ASPH inhibitors MO-I-1151 and MO-I-1182 for 24 h. Confluent cells were scratched with a pipette tip. Cell migration was assessed by measuring the wound area at 0 and 12 h, and the images were quantified using ImageJ software. Magnification: 40×. **(B)** Cells were exposed to 20 μM MO-I-1151 or MO-I-1182 in a transwell migration assay. After 24 h, the fixed and stained cells were quantified based on microscopy images using ImageJ software. DMSO was used as a control. Magnification: 100×. Quantitative data are presented as the mean ± SEM of three independent experiments. The statistical significance refers to the comparison with the control (* p<0.05, ** p<0.01).

To investigate the effect of ASPH inhibitors on the migratory ability of human tumor cell lines, we used a wound healing assay. All the cell lines except Detroit 562 and MCF-7 exhibited significantly reduced migration in response to the influence of the ASPH inhibitors MO-I-1151 and MO-I-1182 (Figure 2A). Although not reaching statistical significance, the migration of the Detroit 562 and MCF-7 cell lines was also lower than that of the corresponding controls. To further evaluate the effect of ASPH inhibitors on migration, we performed a transwell migration assay. As shown in Fig. 2B, treatment with MO-I-1151 and MO-I-1182 resulted in a substantial and statistically significant reduction in migration in all the cell lines tested, including Detroit 562 and MCF-7 cells. The effect of MO-I-1151 was slightly greater in most cell lines.

**Figure 3:**
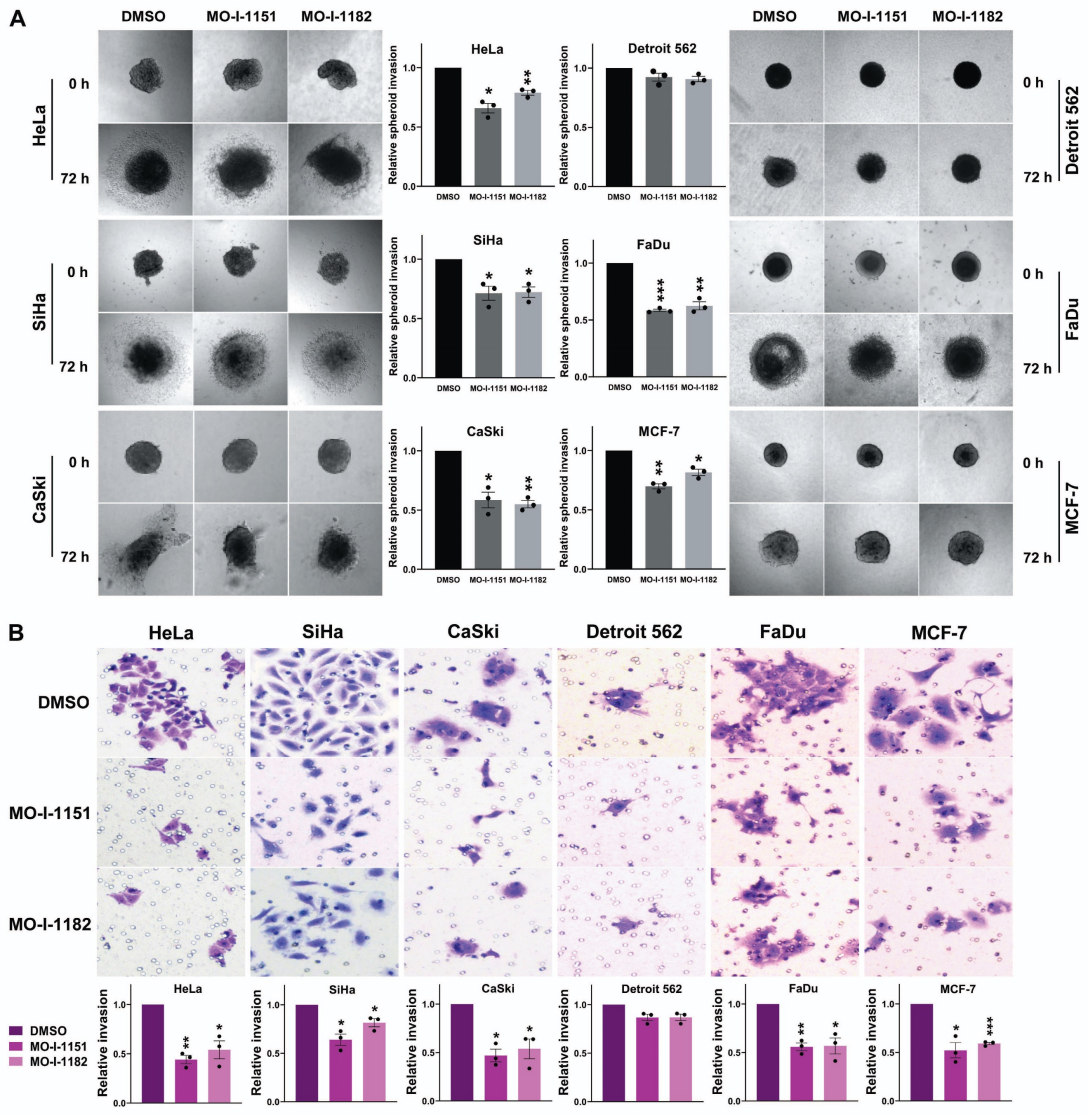
Effect of ASPH inhibitors on cell invasion. **(A)** Cell spheroids were embedded in a 3D collagen matrix and treated with 20 μM MO-I-1151 or MO-I-1182. Images were captured at 0 and 72 h and quantified using ImageJ software. Magnification: 40×. **(B)** Cell lines were exposed to 20 μM MO-I-1151 or MO-I-1182 in precoated Matrigel transwell chambers. After 24 h, the fixed and stained cells were quantified based on microscopy images using ImageJ software. DMSO was used as a control. Magnification: 100×. The data are presented as the means ± SEMs of three independent experiments. The statistical significance refers to the comparison with the control (* p<0.05, ** p<0.01, *** p<0.001).

To assess the effect of the inhibitors on the invasiveness of tumor cells, a 3D spheroid invasion assay was performed. Both the MO-I-1151 and MO-I-1182 treatments had inhibitory effects on the cells compared to the control DMSO-treated cells, which was not significant only in the Detroit 562 cells (Fig. 3A). To validate these findings, we performed a Matrigel-coated transwell invasion assay, providing a more relevant environment for studying invasion. Consistently, all the cell lines except for Detroit 562 exhibited significantly reduced invasion (Fig. 3B). In both invasion assays, MO-I-1151 was more potent in some cell lines (HeLa, MCF-7, SiHa).

### ASPH inhibition affects different signaling pathways in various human tumor cell lines

**Figure 4:**
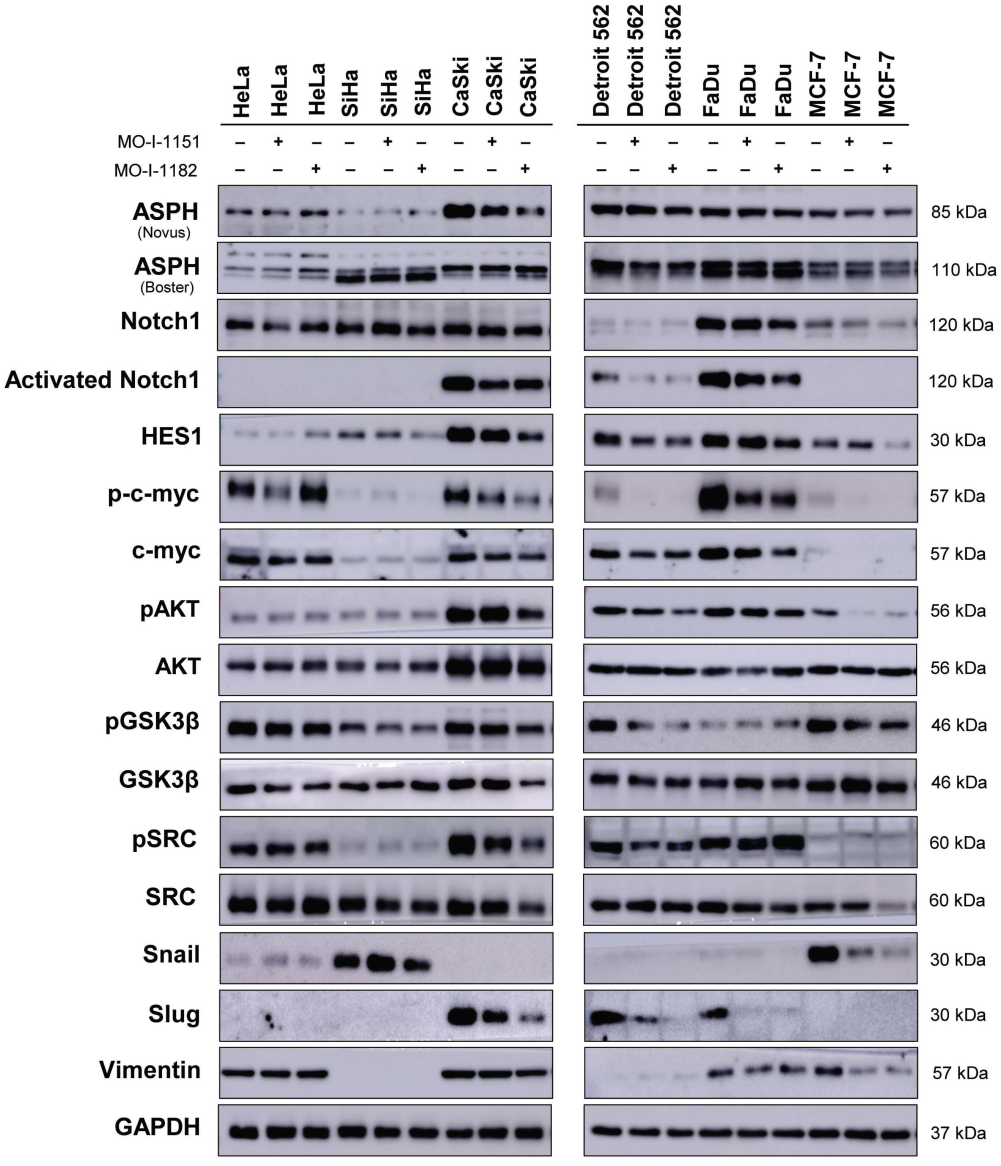
Effect of ASPH inhibitors on cell signaling. The cells were treated with 20 μM MO-I-1151 or MO-I-1182 for 24 h. DMSO was used as an untreated control. Cell lysates were harvested and subjected to SDS‒PAGE and immunoblotting. GAPDH was used as a loading control. The results shown are representative of at least three independent experiments.

To investigate the effect of ASPH inhibitors on cell signaling in human tumor cell lines by western blot (Fig. 4), we first examined ASPH expression using two monoclonal antibodies. While the Novus antibody identifies an epitope at the ASPH N-terminus, the Boster antibody recognizes an epitope at the C-terminus of the full-length ASPH. All the cell lines used in this study produced ASPH, but at varying levels and with different patterns after staining with both the Novus and Boster antibodies.

After treatment with ASPH inhibitors, a decrease in the ASPH level was observed in CaSki cells (detected by the Novus antibody) and Detroit 562 cells (detected by the Boster antibody). The Novus antibody detected ASPH bands at 85 kDa, whereas the Boster antibody detected two ASPH bands at approximately 110 kDa. Since Notch1 signaling is one of the pathways regulated by ASPH-mediated hydroxylation, we examined Notch1 and its downstream targets, HES1 and c-myc. The Notch1 protein was produced in all the tumor cell lines examined, and its level was highly variable. Activated Notch1 was found only in CaSki, Detroit 562, and FaDu cells and its level was reduced by ASPH inhibition. HES1 was also detected in all cell lines, and its level highly varied. High levels of HES1 were found in cell lines with detectable activated Notch1. ASPH inhibition decreased HES1 levels in these cells, and this effect was more pronounced after treatment with MO-I-1182 in the CaSki and FaDu cell lines. Although activated Notch1 was not detected in the SiHa and MCF-7 cell lines, a decrease in HES1 levels was observed in response to MO-I-1182 treatment, corresponding to a reduced level of total Notch1.

c-myc is a critical transcriptional target of the Notch1 pathway 18. In HeLa, CaSki, and FaDu cells, inhibitor-treated samples showed a slight reduction in c-myc levels compared to the respective controls, suggesting that ASPH inhibitors may potentially affect total c-myc expression in these cells. p-c-myc was decreased after treatment with MO-I-1151 in all three cell lines, whereas MO-I-1182 only downregulated p-c-myc expression in CaSki and FaDu cells, and in CaSki cells, this reduction was substantially higher than that after MO-I-1151 treatment. In Detroit 562 cells, a marginal reduction in total c-myc expression was observed only after treatment with MO-I-1151, but p-c-myc was downregulated by both inhibitors. SiHa and MCF-7 cells showed low levels of total c-myc with reduced p-c-myc in SiHa cells after MO-I-1182 treatment and low levels of both p-c-myc and c-myc in MCF-7 cells incubated with both inhibitors. These results suggest that CaSki, Detroit 562, and FaDu cells regulate c-myc expression through the Notch1 pathway.

As in the noncanonical Notch signaling pathway, Notch crosstalks with the PI3K/AKT pathway by inducing AKT phosphorylation to regulate cell fate 19, we also investigated the effect of ASPH inhibitors on the AKT pathway. AKT was expressed at similar levels in all the tumor cell lines examined, except for CaSki cells, which showed higher AKT expression than did all the other cell lines. Treatment with ASPH inhibitors (mainly MO-I-1182) resulted in a decrease of phosphorylated AKT levels in CaSki, Detroit 562, and MCF-7 cells, indicating targeted inhibition of AKT pathway activation in these cell lines. Notably, the reduced AKT phosphorylation in CaSki and Detroit 562 cell lines was consistent with the downregulation of activated Notch1, but HeLa, SiHa and FaDu cells did not exhibit any noticeable alterations in phosphorylated AKT levels despite ASPH inhibition, suggesting a cell line-specific response. These results further highlight the intricate interplay between ASPH, AKT, and Notch signaling.

GSK3β is widely recognized as a downstream target of AKT 20, and previous studies have shown that inhibition of ASPH enzymatic activity leads to increased GSK3β phosphorylation, which promotes tumor cell senescence 21,22. This interaction was also examined in our study, but ASPH inhibitors did not induce GSK3β phosphorylation, suggesting that they may not induce senescence in the cell lines we examined. This assumption was confirmed by X-Gal staining, where no senescence was found after treatment with the inhibitors (data not shown).

A previous study in pancreatic ductal adenocarcinoma showed that ASPH could activate SRC signaling in cancer cells 15. Therefore, we verified the effect of ASPH inhibitors on the SRC pathway. Notably, CaSki and Detroit 562 cells showed a decrease in SRC phosphorylation after treatment with both inhibitors. In MCF-7 cells, inhibition with MO-I-1182 resulted in a decrease in total SRC, while low levels of pSRC remained unaffected. Conversely, the ASPH inhibitor MO-I-1182 induced the upregulation of phosphorylated SRC in FaDu cells. The remaining cell lines did not show a considerable effect on SRC phosphorylation.

As the hydroxylase activity of ASPH is known to upregulate epithelial-mesenchymal transition (EMT) 23, we also examined the expression of EMT-associated markers. ASPH inhibition did not substantially influence vimentin levels in HeLa, CaSki, Detroit 562 and FaDu cells. Vimentin was not detected in SiHa cells, whereas reduced vimentin level was found in MCF-7 cells treated with both inhibitors. Slug, another mesenchymal marker, was downregulated in CaSki, Detroit 562, and FaDu cells after treatment with both ASPH inhibitors, but the HeLa, SiHa, and MCF-7 cell lines did not express Slug. The Snail protein was markedly downregulated only in MCF-7 cells and was undetectable in CaSki and Detroit 562 cells. In HeLa and SiHa cells, the MO-I-1151 and MO-I-1182 inhibitors did not affect any of the detected EMT-related proteins. Our results suggest that ASPH inhibition may alter EMT in part by decreasing Slug expression in CaSki, Detroit 562, and Fadu cells, and vimentin and Snail expression in MCF-7 cells.

### ASPH inhibition affects activated Notch1 localization and nuclear morphology

**Figure 5:**
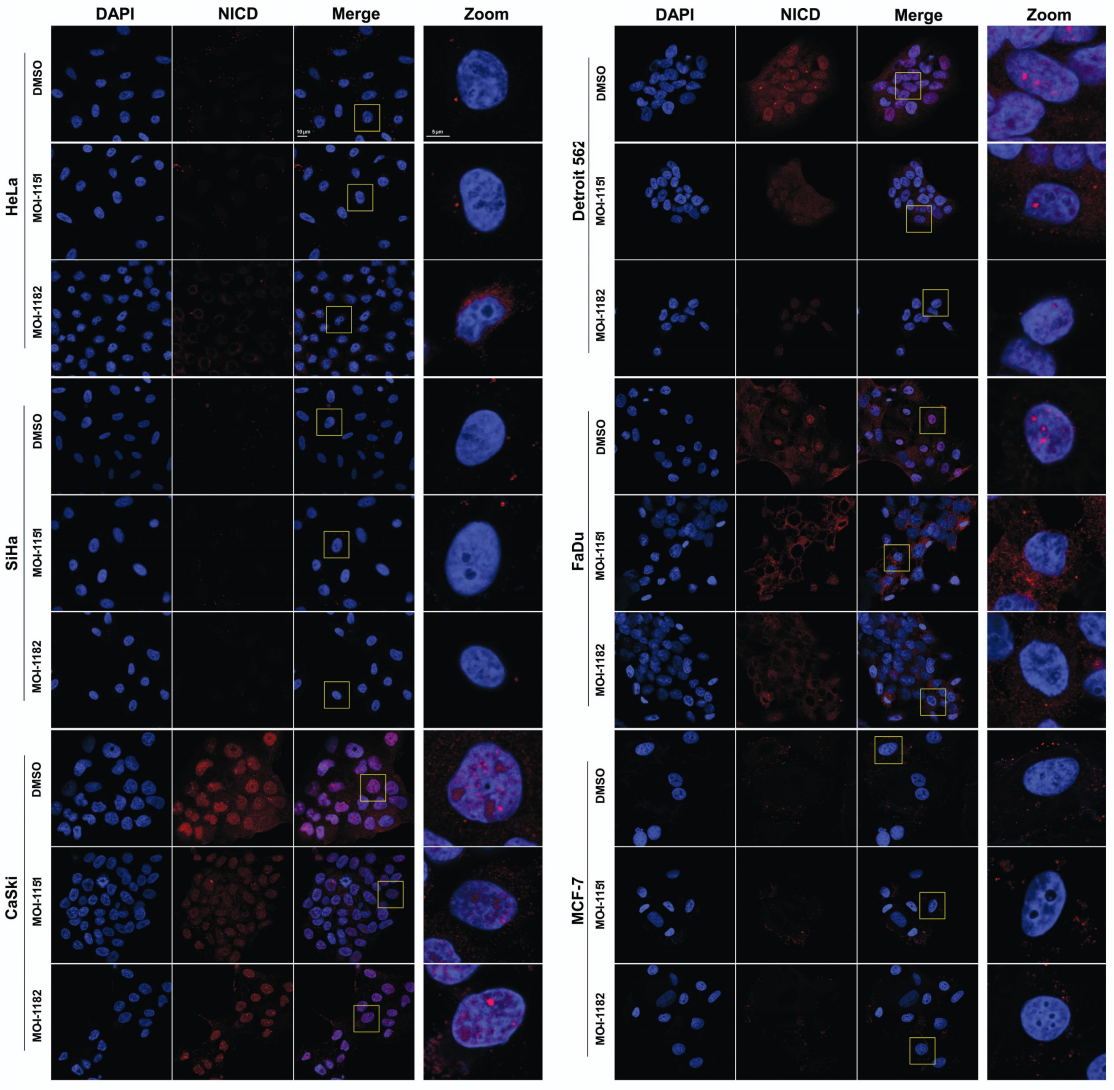
Effect of ASPH inhibitors on activated Notch1 localization. The cells were treated with 20 μM MO-I-1151 or MO-I-1182 for 24 h. DMSO was used as an untreated control. The cells were pre-extracted, fixed with PFA, stained for activated Notch1 and with DAPI, and analyzed via confocal microscopy. Representative images are shown. The scale bars represent 10 μm (Merge) and 5 μm (Zoom).

To validate the immunoblot findings and to explore the subcellular localization of activated Notch1, immunofluorescence detection was performed. In CaSki, Detroit 562, and FaDu cells, activated Notch1 was localized in the nucleus in the form of foci as well as in the cytoplasm, indicating canonical Notch1 activation (Figure 5). Treatment with the MO-I-1151 and MO-I-1182 inhibitors resulted in a significant reduction in both the nuclear and cytoplasmic expression of activated Notch1, corresponding to the immunoblot results showing inhibition of the canonical Notch1 pathway in these cells. Interestingly, changes in nuclear morphology characterized by alterations in the size and/or shape of the nucleus were observed following inhibitor treatment, suggesting a potential link between Notch1 activation and nuclear structure. The absence of activated Notch1 expression in both the immunoblot and immunofluorescence analyses of MCF-7 cells further confirmed that MCF-7 cells do not follow the canonical Notch1 pathway, consistent with the noncanonical Notch1 pathway activation associated with AKT signaling observed in the immunoblot results (Figure 4). No activated Notch1 expression was detected in HeLa and SiHa cells, but ASPH inhibitors affected the morphology of these cells. Notably, MO-I-1182 induced a distinctive effect on nuclear morphology in both cell lines, particularly in HeLa cells, where nuclei showed severe deformation. In addition, SiHa cells treated with MO-I-1151 showed an increase in nuclear size compared to the control. These results in HeLa and SiHa cells indicate that ASPH inhibitors can affect nuclear structure independently of activated Notch1 expression. These findings highlight the diverse effects of ASPH inhibitors on Notch1 signaling and nuclear morphology, providing insight into the impact of these inhibitors on cellular structures and signaling pathways.

### ASPH inhibition induces cell cycle arrest in the G0/G1 phase by downregulating cyclin D1 expression

**Figure 6:**
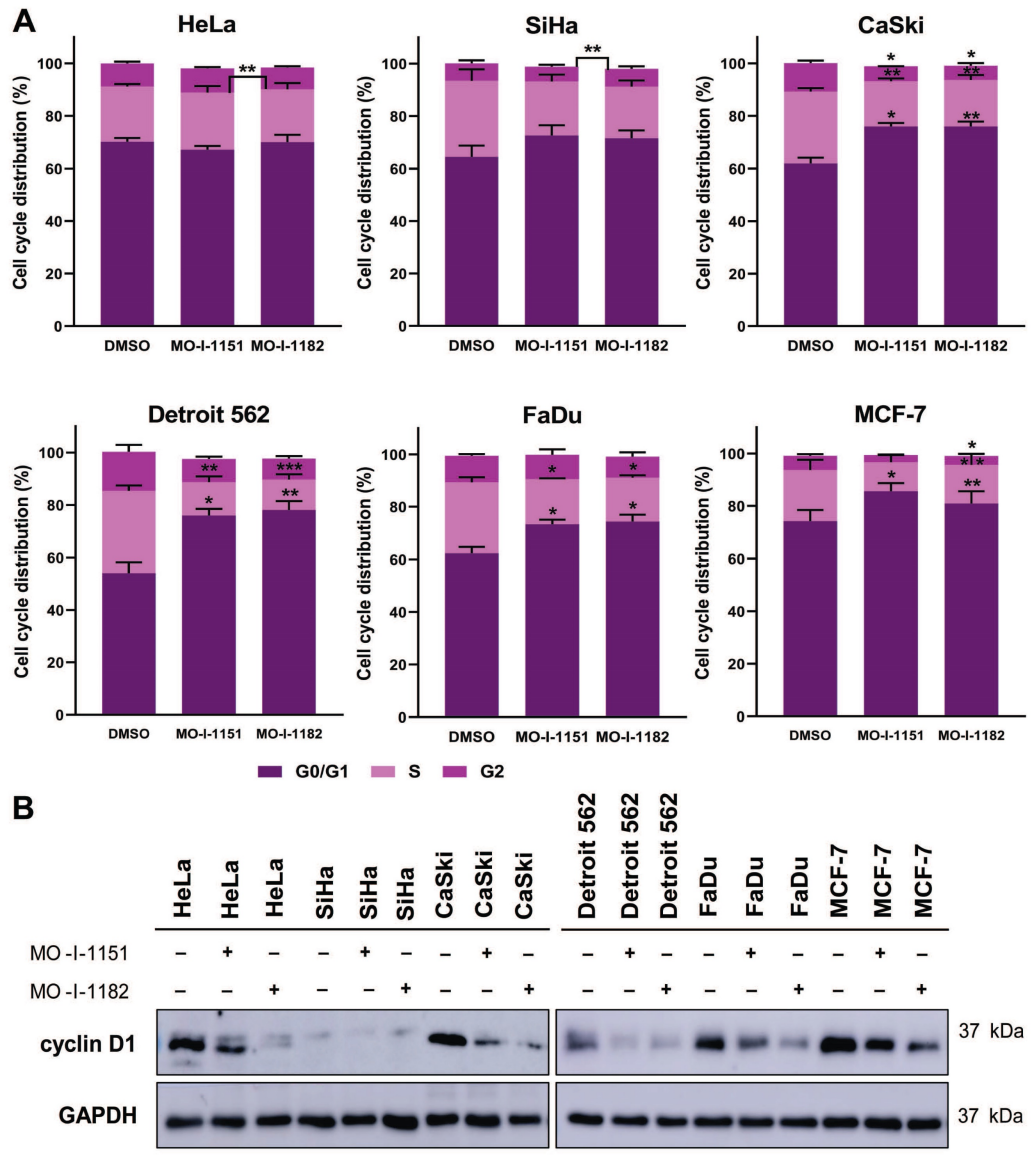
Effect of ASPH inhibitors on cell cycle progression. (A) Cells were treated with 20 μM MO-I-1151 or MO-I-1182 for 48 h. DMSO was used as an untreated control. Cell cycle analysis was performed by flow cytometry with propidium iodide staining. Quantitative data are presented as the mean ± SEM of three independent experiments. The statistical significance refers to the comparison with the control (* p<0.05, ** p<0.01, *** p<0.001). (B) The lysates of cells treated with 20 μM MO-I-1151 or MO-I-1182 were subjected to immunoblot analysis to determine the levels of cyclin D1. DMSO was used as an untreated control, and GAPDH as a loading control. The results shown are representative of at least three independent experiments.

The effect of ASPH inhibitors on cell cycle progression was also examined. The cell lines were exposed to DMSO, MO-I-1151, or MO-I-1182 for 48 h. A reduction in ASPH enzymatic activity caused a marked disruption in cell cycle progression, characterized by an increase in the number of cells in the G0/G1 phase (Figure 6A). In particular, both MO-I-1151 and MO-I-1182 demonstrated significant efficacy in inducing G0/G1 cell cycle arrest in CaSki, Detroit 562, FaDu, and MCF-7 cells. In agreement with these findings, the inhibitors significantly reduced the number of cells in the S phase in these cell lines. Cells in the G2 phase were significantly reduced in CaSki cells with both inhibitors and in MCF-7 cells with MO-I-1182. Notably, HeLa cells did not show G0/G1 arrest but were significantly reduced in S phase with MO-I-1182 compared to MO-I-1151. SiHa cells treated with the MO-I-1151 and MO-I-1182 inhibitors exhibited a notable increase in the percentage of cells in the G0/G1 phase and a decrease in the S phase, although statistical significance was not reached. In addition, SiHa cells showed a significantly higher percentage of cells in the G2 phase after treatment with MO-I-1182 when compared to MO-I-1151 treated cells. Analysis of cell cycle progression showed variable antiproliferative effects of ASPH inhibitors in different tumor cell lines.

To investigate the underlying molecular mechanism of the cell cycle arrest induced by ASPH inhibitors, we detected the level of cyclin D1, a key regulator of cell cycle progression 24. Western blot analysis showed a decrease in cyclin D1 expression after treatment with both ASPH inhibitors in all cell lines except SiHa cells, which exhibited only a slight decrease in cyclin D1 expression upon MO-I-1151 treatment (Figure 6B). MO-I-1182 demonstrated better efficacy than MO-I-1151 in reducing cyclin D1 expression in HeLa, CaSki, FaDu, and MCF-7 cells. These results suggest that ASPH inhibitors may affect cell proliferation by inducing cell cycle arrest in the G0/G1 phase, possibly mediated by cyclin D1 downregulation.

## Discussion

Accumulating evidence has revealed that upregulated ASPH plays a critical role in tumorigenesis 10,11,23,25. The overexpressed ASPH translocated from the endoplasmic reticulum (ER) membrane to the cell surface in cancer cells has correlated with increased cell motility and metastatic potential 26. Furthermore, this translocation makes ASPH more accessible as a potential therapeutic target. Molecular targeted therapy against ASPH using SMIs has recently received considerable attention 6,8,15. ASPH has been shown to directly interact with components of the Notch1 pathway and stimulate this signaling pathway. Therefore, ASPH SMIs can reduce Notch1 signaling and alter the characteristics of cancer cells 27,28. The present study aimed to investigate the effect of ASPH inhibitors on established pathways, with a particular emphasis on the canonical and noncanonical Notch1 signaling pathways in human tumor cell lines of different origins, including cervical (HeLa, SiHa, and CaSki), pharyngeal (FaDu and Detroit 562), and breast (MCF-7) cancer cells. This investigation focused on less studied cell lines in the context of ASPH-mediated tumorigenesis to provide novel insights into the impact of ASPH inhibitors in a broader spectrum of cancer scenarios. The study revealed the diverse and complex behavior of tumor cell lines in response to ASPH inhibition.

To assess the functional impact of ASPH inhibition on the selected cell lines, we used both second-generation (MO-I-1151) and third-generation (MO-I-1182) inhibitors. The third-generation inhibitor MO-I-1182 has improved solubility and has shown greater potency than its second-generation predecessor MO-I-1151 in two previous studies 10,15. Inhibition of ASPH enzymatic activity resulted in significant effects on cell proliferation, migration, and invasion in the various tumor cell lines studied, which was consistent with the results of previous studies 14-16. MO-I-1151 showed more pronounced efficacy than MO-I-1182 in several cell lines, as evidenced by lower IC_50_ values. This is consistent with the study showing greater efficacy of a second-generation ASPH inhibitor than a third-generation inhibitor in colorectal cancer 29.

Initial validation of ASPH synthesis in cell lines by immunoblotting revealed various protein levels, reflecting the intrinsic variability of cellular contexts. This diversity likely contributed to the observed differential responses to the ASPH inhibitors, as ASPH levels were not notably changed in most cell lines but were significantly decreased in CaSki cells and slightly reduced in Detroit 562 cells. These findings highlight the complexity of cellular interactions found in ASPH regulation 3 and emphasize the importance of investigating cell line-specific characteristics to understand the mechanisms governing the differential effects of ASPH inhibitors. Notably, the highest ASPH expression in CaSki, Detroit 562, and FaDu cell lines corresponded to the detection of activated Notch1 solely in these cell lines, where it was reduced after treatment with ASPH inhibitors, similar to the reduction in HES1, a downstream target of canonical Notch signaling 30. Interestingly, in SiHa and MCF-7 cells, where activated Notch1 was not found, a slight reduction in HES1 expression was observed after treatment with the MO-I-1182 inhibitor, suggesting the presence of an alternative signaling pathway that regulates HES1 independently of activated Notch1. However, this observation could also be due to the low sensitivity of detecting activated Notch1 in these cells. Notch modulation by ASPH has been well characterized in neuroblastoma 31, hepatocellular carcinoma 32, and prostate 21, pancreatic 10, and breast cancers 11. A study using a first-generation ASPH inhibitor (MO-I-1100) reported findings consistent with our results, linking growth reduction in pancreatic cancer to downregulation of the Notch signaling pathway 10. In exploring the interplay between ASPH and Notch signaling in human tumor cells of diverse origins, our study is also consistent with the findings of Barboro et al. 21, Lin et al. 11, and Lawton et al. 32, where disruption of the Notch signaling pathway was similarly observed upon ASPH downregulation. Our study contributes to this understanding by examining the effect of ASPH inhibition on the Notch1 pathway in various tumor landscapes. To further explore the role of the Notch1 signaling pathway, our study examined the noncanonical cascade of the Notch1 pathway, which functions independently of the CSL complex and is activated either by the binding of noncanonical ligands to the Notch1 receptor or in the absence of ligands 12,33. Since Notch signaling promotes malignant progression by activating the PI3K/AKT pathway 34, we sought to determine whether the effect of ASPH inhibitors is associated with an alteration in the phosphorylation status of the Ser473 AKT in the PI3K/AKT pathway in our selected cell lines. Interestingly, treatment with the MO-I-1182 inhibitor resulted in reduced Ser473 AKT phosphorylation in CaSki and Detroit 562 cells, despite the occurrence of active canonical signaling in these cell lines. These results likely represent a complex regulatory network that may involve feedback loops and crosstalk with canonical Notch signaling pathway. Moreover, both ASPH inhibitors markedly reduced the Ser473 AKT phosphorylation in the MCF-7 cell line, which corresponds to the findings of studies highlighting the impact of ASPH expression on cellular processes via the MAPK/ERK and PI3K/AKT pathways 19,35.

Previous studies have shown that the activated Notch1 protein forms distinct nuclear foci 36,37, indicative of regions characterized by increased transcriptional activity 38. Based on our immunoblot analyses revealing altered activated Notch1 expression after treatment with ASPH inhibitors, we predicted that treatment with these agents would affect the nuclear localization of activated Notch1. Subsequent immunofluorescence analysis not only validated our prediction but also provided new findings. In cells with canonical Notch1 activation (CaSki, Detroit 562, and FaDu), the considerable reduction in nuclear and cytoplasmic activated Notch1 expression following inhibitor treatment confirmed the inhibitory effect on the canonical Notch1 pathway. The concomitant changes in nuclear morphology suggested a potential link between Notch1 activation and structural alterations in the nucleus. Interestingly, the cell lines lacking detectable activated Notch1 expression also exhibited changes in nuclear morphology upon treatment with ASPH inhibitors, suggesting that ASPH inhibitors have a broader influence on cellular structures beyond Notch1 signaling. While several studies have reported the effect of chemotherapeutic drugs on nuclear structure, the exact mechanism behind these alterations is largely unknown 39-41. Our study, which is the first to report alterations in nuclear structure upon treatment with ASPH inhibitors, underscores the need for further investigation to understand the underlying mechanisms involved.

Activation of the ASPH-SRC axis is a key driver of tumor progression in pancreatic 15 and breast cancer 39. Our results showed a notable decrease in SRC phosphorylation after ASPH inhibition only in CaSki and Detroit 562 cells, although phosphorylated SRC was detected in all the cell lines tested, with high levels in HeLa, CaSki, Detroit 562, and FaDu cells. Reduced SRC phosphorylation was associated with a decrease in canonical Notch1 signaling in CaSki and Detroit 562 cells. These results, showing heterogeneity in the response to ASPH inhibition, further indicate the complex involvement of ASPH in the cellular regulatory network.

Furthermore, ASPH has been reported to stimulate EMT through interactions with the vimentin, ERK/MAPK, and PI3K/AKT pathways 23,35. In the cell lines exhibiting canonical Notch1 signaling (CaSki, Detroit 562, and FaDu), Slug was expressed, and it was downregulated by ASPH inhibition. Conversely, in MCF-7 cells with an activated noncanonical Notch1 pathway, ASPH inhibition led to the downregulation of Snail and vimentin. This difference in the response between the canonical and noncanonical Notch signaling pathways highlights the diverse effects of ASPH inhibition on different cell lines. The downregulation of Snail, Slug, and vimentin in response to ASPH inhibition, as observed in the present study, corresponds to the suppression of key molecular markers associated with EMT. The effect of ASPH inhibitors on EMT-associated markers is consistent with previous findings in which ASPH inactivation strategies, such as targeting with miR-200a 35, knocking out with CRISPR/Cas9 15,25, silencing with shRNA 23, or disrupting catalytic activity with SMI 15 have demonstrated EMT suppression in cancer cells. These results suggest that the influence of ASPH inhibition extends beyond canonical Notch signaling and may involve alternative pathways or crosstalk mechanisms.

The SiHa cell line exhibited minor dysregulation of the pathways examined after ASPH inhibition, except for HES1 and Snail downregulation after treatment with MO-I-1182. This differential response, characterized by a lack of a significant effect on the studied pathways in SiHa cells, may be due to the comparatively lower ASPH expression or other contributing factors in this cell line, highlighting the complexity of cellular responses and the possible involvement of another pathway in this context.

As we found reduced cell proliferation after ASPH inhibition, we also analyzed the effect of the ASPH inhibitors on cell cycle progression. Cyclin D1, which is regulated by the Notch 43 and PI3K/AKT signaling pathways 44, is a key protein in the G1 phase of the cell cycle and is associated with high proliferative activity 45. The cell transition from G1 to S phase is promoted by c-myc, another target of Notch1 signaling. Reduced ASPH activity may inhibit cell cycle progression by suppressing cyclin D1 and c-myc expression 14,35. We showed the suppressive effect of ASPH inhibitors on cyclin D1 expression, c-myc phosphorylation, and the transition from G1 to S phase in the examined tumor cell lines. We assumed that ASPH inhibitors can reduce cyclin D1 expression via a Notch1-dependent canonical pathway (CaSki, Detroit 562, and Fadu cells) or a noncanonical pathway mediated by PI3K/AKT signaling (MCF-7 cells). In HeLa and SiHa cells, where activation of Notch1 or SRC signaling was not detected after ASPH inhibition, a different pathway is likely to apply.

The study demonstrated the heterogeneous response of different cell lines to ASPH inhibition. This heterogeneity was manifested in the effect on (i) cell proliferation, migration, and invasion; (ii) activation of the canonical and noncanonical Notch1 signaling pathways; (ii) nuclear localization of activated Notch1; (iii) nuclear morphology; (iv) activation of the SRC signaling pathway; (v) levels of proteins involved in EMT; and (vi) cell cycle progression. Intrinsic characteristics of the cancer cell lines studied related to their histological origin, genetic instability leading to mutagenesis in various oncogenic pathways, and ASPH levels may be important sources of heterogeneous response to ASPH inhibition. Such heterogeneity could be predicted from previous studies on the role of ASPH in carcinogenesis when different pathways driven by ASPH were individually identified in different cell lines 3,46,47. These pathways may be inhibited (GSK-3β 16) or activated (Notch1 10, SRC 15) by ASPH in a cell type-specific manner, leading to heterogeneous responses to ASPH inhibition in different cancer cell lines. However, while these studies lack comprehensive insight into the heterogeneous response to ASPH inhibition because they primarily focused on a single cancer type, and a limited number of signaling pathways, our study compared 6 cell lines representing 3 cancer types and performed a complex characterization of cellular processes affected by ASPH inhibition, including novel analyses of activated Notch1 localization, nuclear morphology, and cell cycle progression.

## Conclusion

The Notch signaling pathway plays a critical role in the initiation and progression of human cancers. Our study elucidated the heterogeneous responses of various tumor cell lines to ASPH inhibition, which are mediated either by suppressing the activation of the Notch1 receptor, resulting in a reduction in NICD translocation to the nucleus and downregulation of downstream targets of the canonical Notch1 pathway, or by affecting the noncanonical Notch1 pathway by targeting AKT phosphorylation. Through canonical and noncanonical pathways, ASPH inhibitors interrupt cell cycle progression by downregulating cyclin D1 and arresting cells in the G0/G1 cell cycle phase, thereby inhibiting the proliferation of cancer cells. This study highlights the complex nature of ASPH-mediated regulation, involving not only the Notch1 pathway but also diverse pathways in different cancer types, and emphasizes the importance of targeting multiple pathways to reduce cancer progression. Understanding the precise mechanisms by which ASPH inhibitors modulate cell signaling in tumor cells may pave the way for improved cancer treatment.

## Abbreviations

ADAM12/15: A disintegrin and metallopeptidase domain 12/15
ASPH: Aspartate β-hydroxylase
BSA: Bovine serum albumin
c-myc: c-myelocytomatosis oncogene
DAPI: 4,6-diamidino-2-phenylindole
DMEM: Dulbecco's modified Eagle's medium
DMSO: Dimethyl sulfoxide
EDTA: Ethylenediaminetetraacetic acid
EMEM: Eagle's minimum essential medium
EMT: Epithelial-mesenchymal transition
ER: Endoplasmic reticulum
ERK: Extracellular signal-regulated kinase
FBS: Fetal bovine serum
GAPDH: Glyceraldehyde-3-phosphate dehydrogenase
GSK-3β: Glycogen synthase kinase-3β
HCC: Hepatocellular carcinoma
HES1: Hes family bHLH transcription factor 1
HPV-16: Human papillomavirus type 16
HRP: Horseradish peroxidase
MAPK: Mitogen-activated protein kinase
mTORC2: Mechanistic target of rapamycin kinase C2
MTT: 3-[4,5-dimethylthiazol-2-yl]-2,5-diphenyl-tetrazolium bromide
NFκB: Nuclear factor kappa B
NICD: Notch intracellular domain
PBS: Phosphate-buffered saline
PFA: Paraformaldehyde
PI: Propidium iodide
PI3K/AKT: Phosphatidylinositol-3-kinase/protein kinase B
PTEN: Phosphatase and tensin homolog
PVDF: Polyvinylidene difluoride
RPMI-1640: Roswell Park Memorial Institute 1640
SDS: Sodium dodecyl sulfate
shRNA: Short hairpin RNA
SMI: Small molecule inhibitor
SRC: SRC tyrosine kinase
STAT-3: Signal transducer and activator of transcription 3
TGF-β: Transforming growth factor β
